# Treatment-related mortality in childhood cancer patients – who, when and how much

**DOI:** 10.2340/1651-226X.2024.40315

**Published:** 2024-07-01

**Authors:** Trausti Oskarsson, Fredrik Baecklund

**Affiliations:** a Pediatric Oncology, Astrid Lindgren Children’s Hospital, Karolinska University Hospital, Solna, Sweden; b Pediatric Oncology, Department of Women’s and Children’s Health, Karolinska Institutet, Solna, Sweden

**Keywords:** Childhood cancer, treatment-related mortality

Treatment-related mortality is a dreadful complication of childhood cancer treatment. During 2003–2012 it was estimated to affect 3.9–4.6% of children diagnosed with cancer in high-resource countries [1–3]. The proportion of all childhood cancer deaths that can be attributed to treatment toxicity has increased over time as the number of cancer-related deaths have decreased [4]. The most common causes of treatment-related death are infection, bleeding, and organ failure [1–3]. It is more common among children treated according to intensive chemotherapy protocols and those undergoing allogeneic hematopoietic stem cell transplantation (HSCT) [1–3]. Whether the absolute proportion of treatment-related deaths has changed over time is less clear, especially in more recent years.

Sørensen and colleagues conducted a cohort study with data from the Danish Childhood Cancer Registry [5] to investigate the frequency of treatment-related mortality among 3,255 children (0–14 years old) diagnosed with cancer during 2001–2021. The primary aim was to compare treatment-related mortality within 5 years from diagnosis between two time periods, 2001–2010 and 2011–2021, and to see if the overall improvement in survival observed among childhood cancer patients over the last decades in part could be explained by reduced treatment-related mortality. All treatment-related deaths were counted, irrespective of line of treatment, so the estimated incidence concerns the whole cancer journey, as most childhood cancer patients will be either cured or dead 5 years after primary diagnosis. They found that the proportion of treatment-related death within 5 years from diagnosis in the whole cohort was fairly low in both time periods, 3.3% versus 2.5%. Although the absolute number was lower in the later period, the difference was not statistically significant (*p* = 0.20). The pattern was similar for the three main groups of childhood cancer: central nervous system (CNS) tumors (2.3% vs. 1.5%), hematological malignancies (5.9% vs. 4.3%) and solid tumors outside the CNS (0.9% vs. 1.3%). The main causes of treatment-related death were infection (37%), nervous system complications (14%), and hemorrhage (11%), which is in line with previous studies [1–3]. Because the frequency of treatment-related death has been stable over the last couple of decades, the observed increased survival among childhood cancer patients reasonably must be explained mainly by an increase in cure – a conclusion that was supported by the present data (death from progressive cancer decreased from 12.0 to 9.7%, *p* = 0.02).

Although it is reassuring that death due to cancer treatment is uncommon, every such event is one too many. Knowing which patients and timepoints are associated with the greatest risk of treatment-related death could help identify risk situations.

In exploratory analyses, the study team identified patient (infants and females) and disease characteristics (hematological malignancies, advanced disease, relapsed cancer), and treatment-related factors (early treatment phases, allogeneic HSCT) associated with treatment-related mortality. These factors differed somewhat between cancer types. Among children with CNS tumors, young age at diagnosis was borderline significantly associated with treatment-related mortality. Among children with hematological malignancies, associated factors were sex, age at diagnosis, CNS involvement, relapsed cancer, and HSCT (borderline significant). Among children with solid tumors, having metastatic disease at diagnosis and HSCT were associated with treatment-related mortality. As the authors point out, these high-risk groups need specific measures to reduce toxicities without impairing the chance of long-term remission.

In hematological malignancies, recent trials have been successful in simultaneously improving treatment response and reducing treatment-related mortality. For example, the addition of tyrosine kinase inhibitors to the treatment of children with BCR::ABL1-positive acute lymphoblastic leukemia (ALL) has reduced the indication for allogeneic HSCT [6], and replacing intensive chemotherapy with less toxic alternatives has improved outcomes of relapsed childhood B cell ALL (blinatumomab) [7] and acute promyelocytic leukemia (arsenic trioxide) [8]. Examples of ongoing attempts are the ALLTogether (NCT03911128) and Interfant-21 trials (NCT05327894), in which chemotherapy is replaced by immunotherapy in subgroups of patients with Down syndrome-associated ALL and KMT2A-rearranged infant ALL, respectively, both of whom are examples of patient groups especially vulnerable to the toxic effects of conventional chemotherapy. Among children with B cell malignancies, where new effective and less toxic treatment regimens exist or are being evaluated, we can expect to see a reduction in treatment-related death in the coming years.

When less toxic treatment options are not (yet) an alternative, which is still the case in most T-cell malignancies and solid tumors, it is important to be able to identify risk situations to take preventive measures, if possible, and to stay vigilant. In addition to who are at the greatest risk, there is the question of at what time point during treatment the risk of treatment-related death is the greatest. In this study, most treatment-related deaths occurred during the first 3 months after diagnosis and after relapse. These are situations when the tumor burden is high and may impair organ functions. The authors speculate (and provide supporting references), that starting cancer treatment adds weight to the burden, which could explain why the risk of fatal complications is the greatest in these situations. The data from this study along with those of previous ones [1–3] are helpful in identifying in which patients and at which time point particular attention to treatment-related death is warranted.

Sørensen and colleagues defined treatment-related mortality according to a classification system for children with cancer proposed by the International Paediatric Oncology Mortality Classification Group (IPOMCG), an international group of experts in supportive care in childhood cancer from the United States, Canada and Europe [4]. The use of a common classification in epidemiological studies is important, as it allows for comparison between studies and over time, and is thus a strength of this study. Another strength is the high-quality data provided by the Danish Childhood Cancer Registry, a nationwide quality registry that includes all children 0–14 years old diagnosed with cancer [5]. The results should thus be valid and possible to extend to similar cohorts of childhood cancer patients in other high-resource countries. Despite a reasonably large cohort of childhood cancer patients, the rare outcome measure resulting in few events limited precision and power in certain subgroup analyses, and limited the number of subgroup analyses that could be performed (for instance for specific diagnoses).

To reduce treatment-related mortality in the future, key areas for development are treatment de-intensification for patients at low risk for relapse, implementation of novel therapies and improved supportive care. Further implementation of precision medicine in clinical practice will provide new opportunities for risk stratification and individualized targeted therapies. Sequencing of the host genome at diagnosis has the potential to unveil germline variants associated with excess treatment toxicity and to modify the treatment accordingly. Another aspect of treatment-related mortality, which was not the subject of this study, is the observed excess late mortality among adult childhood cancer survivors. As the number of adult survivors of childhood cancer is increasing, data on late mortality is expanding. In this setting, the main treatment-related causes of death are second malignancies and cardiovascular events [9]. Fortunately, following more restricted use of therapeutic radiation and decreased exposure to anthracyclines during the last decades, a reduction in late mortality has been seen [10]. Above-mentioned areas of development also have the potential to reduce late mortality of childhood cancer treatment.

On a global level, there are great disparities in treatment-related mortality between high-income countries and low- and middle-income countries. Although some improvements have been observed in middle-income countries, fatal toxicities are still a major problem in low-income countries [11]. It is a multifactorial problem, where major contributing factors are scarce resources on all levels (country, hospital, family) and underdeveloped infrastructure, limiting the access to healthcare overall and in particular the highly specialized care needed to treat childhood cancer patients, including dedicated pediatric oncologists and nurses, anti-cancer drugs, and supportive care to prevent and treat infections and other complications, intensive care units for children, blood banks and more [11]. Improvements on all these levels are urgently needed to reduce the global disparities in treatment-related mortality in childhood cancer patients.

In conclusion, Sørensen and colleagues provide evidence that the risk of treatment-related mortality remains low among children treated for cancer in a high-resource country. Together with previous studies, their work can help us identify risk patients and situations with the potential to prevent some treatment-related deaths. To reach null treatment-related mortality in the future, new ways of treating childhood cancer, further developed supportive care and equal access to good healthcare across the world are needed.

## Data Availability

No data were used to write this editorial.
